# Evaluation of the effect of clinical characteristics and intensive care treatment methods on the mortality of covid-19 patients aged 80 years and older

**DOI:** 10.1186/s12871-021-01511-6

**Published:** 2021-11-22

**Authors:** Sibel Oba, Mustafa Altınay, Aysel Salkaya, Hacer Şebnem Türk

**Affiliations:** Department of Anesthesiology and Reanimation, Şişli Hamidiye Etfal Training and Research Hospital, Halaskargazi caddesi, 34371, Şişli, Istanbul, Turkey

## Abstract

**Background:**

Older adults have an increased risk of mortality from Coronavirus disease 2019 (Covid-19). Despite the high number of publications on the topic of Covid-19 pandemic, few studies have focused on the intensive care treatments of Covid-19 patients aged 80 years and older.

The goal of our study is to investigate the effect of the intensive care treatments on the mortality of Covid-19 patients aged 80 years and older based on their clinical features, laboratory findings and the intensive care treatments methods.

**Methods:**

The data of 174 patients aged 80 years and older treated from Covid-19 in intensive care unit were assessed retrospectively. The patients were divided into two groups as survivor and non-survivor. The effects of age, gender, length of stay, comorbid diseases, laboratory values, thoracic computed tomography findings, having invasive mechanical ventilation (IMV), high flow nasal cannula (HFNC) and/or non-invasive mechanical ventilation (NIMV), hemodiafiltration (HDF), anti-cytokines and plasma therapy on mortality have been investigated.

**Results:**

The mean age and mean values of CRP, PCT, Ferritin, LDH were statistically significantly high in the non-survivor group. The mortality rate of the patients who had IMV was also statistically significantly higher compared to patients who had HFNC and/or NIMV. Albumin level and the rate of treatment with HFNC and/or NIMV were statistically significantly low in non-survivor group compared to the Survivor group.

**Conclusion:**

ICU treatments may be beneficial for the Covid-19 patients aged 80 years and older. Increased age, high levels of CRP, PCT, ferritin, and having IMV are detected as poor outcome markers.

## Background

During the Coronavirus disease 2019 (Covid-19) pandemic, older adults have been the most vulnerable age group. Data from international intensive care experience indicate that patients older than 65 years of age are more likely to require hospitalization, admission to intensive care and had a higher mortality rate [1, 2]. However, few reports have investigated the clinical course and characteristics of Covid-19 among the elderly patients aged 80 years and older [3–5]. Moreover these reports were mostly about the mortality and morbidity of 80 years and older Covid-19 patients treated in local hospitals without intensive care unit (ICU) or in nursing homes [6].

Despite the high number of publications on the topic of Covid-19 pandemic, there are various issues about the treatment of the disease yet to be resolved. Among such issues, is the prognosis of Covid-19 patients older than 80 years with respiratory failure who require ICU treatments. There is a limited number of studies about the intensive care treatments of Covid-19 patients older than 80 years. The results of these studies are controversial: some of the researchers have concluded that the mortality rate among patients 80 years and older who required advanced therapies in intensive care is very high and that invasive intensive care treatment fails to save the lives of the vast majority of elderly patients with respiratory insufficiencies [4]. On the other hand, certain previous studies have revealed that chronological age alone should never be the sole criterion for intensive care treatment indication and there are some older patients who may receive greater benefit from ICU care than younger patients [5].

The Covid-19 pandemic is pressuring health care systems. Many countries have adopted a decision guide about the use of medical resources such as ventilators or dialysis machines in order to avoid the collapse of ICUs and to maximize the health care benefits [7]. However, during this period of Covid-19 pandemic there has been no triage or selection process applied by Turkish authorities and every Covid-19 patient in need of intensive care treatment was admitted to public hospitals. Consequently, a significant data about Covid-19 patients aged 80 years and older who received intensive care treatment was collected. In this retrospective study, the efficacy of the ICU treatments on the mortality of Covid-19 patients aged 80 years and older is investigated based on their clinical features, laboratory findings and the intensive care treatments methods.

## Methods

This study was approved by the ethics committee of Şişli Hamidiye Etfal Training and Research Hospital with approval number 02.03.2021/1823. All methods were carried out in accordance with Declaration of Helsinki. Data were collected retrospectively about patients 80 years and older treated from Covid-19 in Şişli Hamidiye Etfal Training and Research Hospital ICU between March 1st, 2020, and January 31st, 2021.

We included all patients with age > 80 years who received ICU treatment for Covid-19. We excluded patients who were lacking data.

Şişli Hamidiye Etfal Hospital is one of the largest academic hospitals in Turkey. During the Covid-19 pandemic period, five ICUs with 45 beds in total were rapidly created for the treatment of patients. All patients with nasal cannula or reservoir mask were followed up in another unit in emergency department and the patients in need of intubation or high flow nasal cannula (HFNC) (respiratory rate > 30/min, oxygen saturation < 94%, PaO_2_/FiO_2_ < 300) were admitted to ICU. No selection criteria (such as old age or comorbidity) were applied and all Covid-19 cases were managed according to international treatment guidelines. The patients were divided into two groups as survivor and non-survivor. The relevant data was obtained from our hospital’s electronic information system and discharge reports of the patients. This data included, age, gender, length of stay, additional diseases (such as Diabetes Mellitus (DM), hypertension (HT), coronary artery disease (CAD), chronic obstructive pulmonary disease (COPD) and other diseases), baseline laboratory values at the induction to ICU (C-reactive protein (CRP), procalcitonin (PCT), Ferritin, D-dimer, Troponin, Lactate, Albumin, Urea, Creatinine, ALT, AST, PaO_2_, PaCO_2_, lactate dehydrogenase (LDH), WBC and Lymphocyte. Covid-19 diagnoses were confirmed using polymerase chain reaction testing of nasopharyngeal samples (SARS-CoV2 RT-PCR(+)) and based on the thoracic computed tomography (CT) of the patients. Thoracic CT findings of the patients at the time of admission to the ICU were classified as no-infiltrate, unilateral infiltrates and bilateral infiltrates. The invasive treatment methods investigated were invasive mechanical ventilation (IMV), high flow nasal cannula (HFNC) and/or non-invasive mechanical ventilation (NIMV), hemodiafiltration (HDF), anti-cytokines and plasma therapy.

### Statistical analysis

Statistical analysis of the data was performed through the Windows SPSS15.0 program. Descriptive statistics were given in terms of number and percentage for the categorical variables and in terms of median and interquartile range (IQR) for the numeric variables. The rates in groups were compared via the chi-squared test. Comparisons of two independent groups were made with the Mann Whitney U test, since the numerical variables did not follow the normal distribution condition. The prognosis power of the inflammatory parameters to predict mortality was assessed based on the analysis of the Receiver Operating Characteristic (ROC) curve. Determining factors were investigated with the Logistic Regression Analysis. Statistical alpha significance level was determined at *p* < 0.05.

## Results

A total of 174 patients 80 years and older were included in the study. Demographic and clinical characteristics of patients were presented comparatively as survivor and non-survivor groups in Table 1. Twenty-four patients (13.8%) were older than 90 years old. Mean age of the non-survivor group was 86 and was statistically significantly high compared with the survivor group (*p *= 0.037).

**Table 1:** Demographic and clinical characteristics of the patient cohort

			TOTAL (*N* = 174)	SURVIVOR *n* = 34 (%19.5)	NON-SURVIVOR *n* = 140 (%80.5)	*P*
**DEMOGRAHY**	Age Median (IQR)		85 (81-89)	83 (80.75-86.5)	86 (82-89)	***0.037***
	Age	80-90	150 (86.2%)	31 (91.2%)	119 (85.0%)	0.420
		> 90	24 (13.8%)	3 (8.8%)	21 (15.0%)	
	Sex	Male	102 (58.6%)	21 (61.8%)	81 (57.9%)	0.678
		Female	72 (41.4%)	13 (38.2%)	59 (42.1%)	
	Length of Stay		7 (3.75-12)	7 (4-11.25)	7 (3-12.75)	0.976
**COMORBIDITY**	DM		56 (32,2%)	11 (32.4%)	45 (32.1%)	0.981
	HT		100 (57.5%)	21 (61.8%)	79 (56.4%)	0.572
	CAD		52 (29.9%)	10 (29.4%)	42 (30.0%)	0.946
	COPD		31 (17.8%)	8 (23.5%)	23 (16.4%)	0.332
	Other		115 (66.1%)	19 (55.9%)	96 (68.6%)	0.161
**LABORATORY VALUES** Median (IQR)	CRP		120.5 (69-202.25)	87.5 (35.75-183.25)	124 (76.25-210.75)	***0.011***
	PCT		0.61 (0.23-2.76)	0.295 (0.13-1.475)	0.725 (0.28-3.185)	***0.030***
	Ferritin		434 (189-952)	239 (89.35-491.5)	568 (232.25-1072.5)	***0.002***
	D-Dimer		1549.5 (868.5-3281.75)	1280 (824.5-2527)	1620 (897.5-3407)	0.274
	Troponin		7 (0.07-81.6)	13 (0.23-47.25)	0.66 (0.065-88)	0.426
	Lactate		1.93 (1.4-2.95)	1.785 (1.23-2.595)	2.015 (1.425-3.15)	0.128
	Albumin		27 (21.9-31.8)	29.55 (23.6-34.15)	26.5 (21.5-30.8)	***0.032***
	Urea		76 (55.75-118.5)	64.5 (50.25-97.5)	77.5 (59-120.75)	0.096
	Creatinine		1.235 (0.86-2.045)	1 (0.74-1.78)	1.32 (0.92-2.14)	0.074
	ALT		21 (14-35)	22.5 (13.75-32)	20.5 (14-35.75)	0.894
	AST		38 (25-55)	32.5 (23.75-43.25)	40 (25-61)	0.096
	PaO_2_		78 (60-104)	84 (59-117.25)	78 (60-100)	0.530
	PaCO_2_		36 (28-49.25)	34.2 (28.75-50)	37 (28-49)	0.953
	LDH		362 (270.5-517.5)	285 (233.75-486.75)	381 (292-536)	***0.019***
	WBC		11,140 (7085-15,395)	10,762 (6927.5-14,017.5)	11,600 (7150-16,090)	0.365
	Lymphocyte		690 (440-1165)	855 (535-1550)	660 (420-1130)	0.078
**TREATMENT**	IMV		141 (81.0%)	5 (14.7%)	136 (97.1%)	***< 0.001***
	HFNC and/or NIMV		33 (19%)	29 (85.3%)	4 (2.9%)	
	HDF		10 (5.7%)	4 (11.8%)	6 (4.3%)	0.107
	Anti-cytokines		3 (1.7%)	1 (2.9%)	2 (1.4%)	0.481
	Plasma		5 (2.9%)	2 (5.9%)	3 (2.1%)	0.252
**COMPUTED TOMOGRAPHY**	Non-Infiltration		32 (18.4%)	10 (29.4%)	22 (15.7%)	0.147
	Unilateral Infiltration		23 (13.2%)	5 (14.7%)	18 (12.9%)	
	Bilateral Infiltration		119 (68.4%)	19 (55.9%)	100 (71.4%)	

**Fig. 1:**
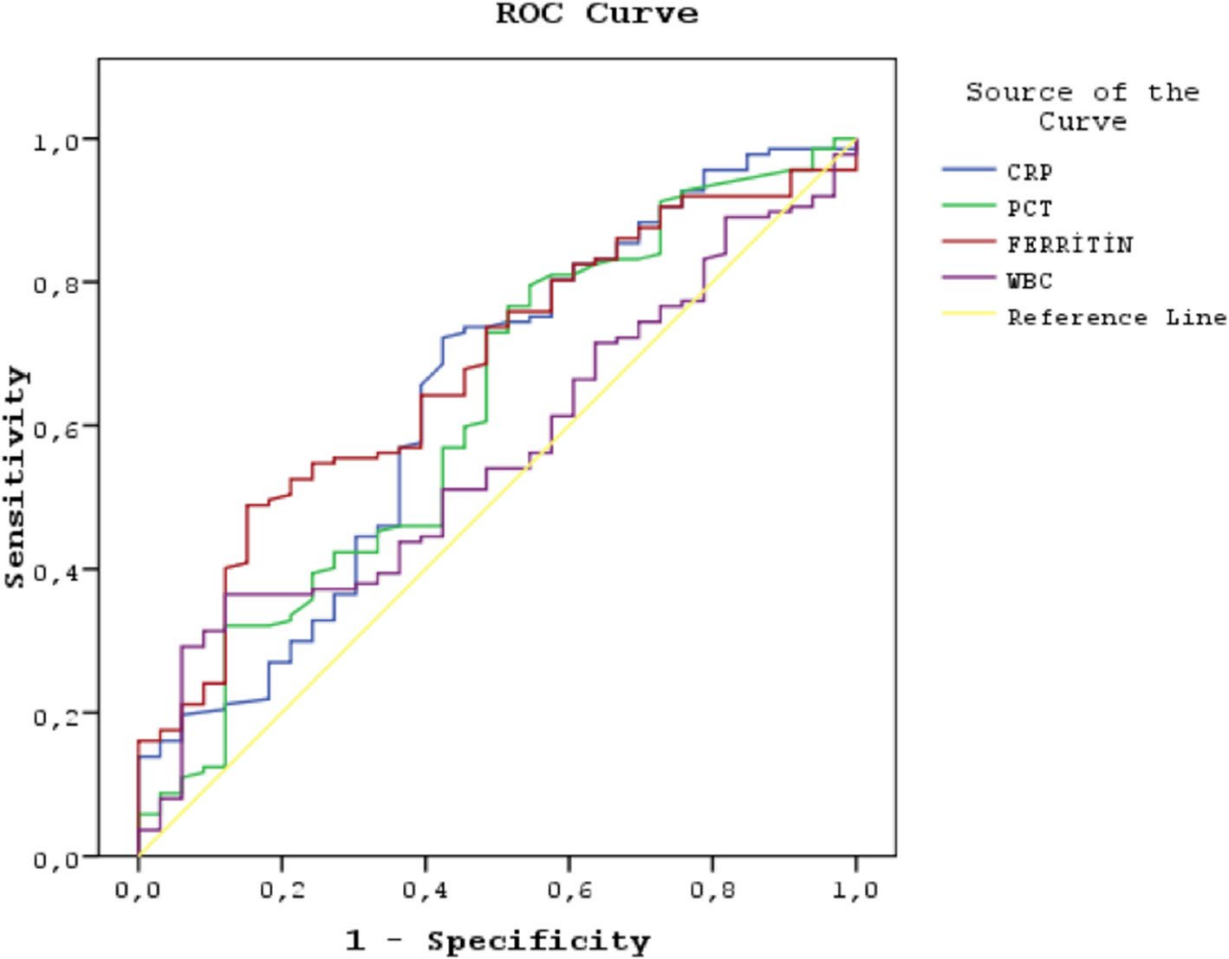
Inflammatory parameters in ROC curve

Bold and italic values are statistically significant (*p* < 0.05)

group (*p* = 0.037). The mean CRP (*p* = 0.011), PCT (*p* = 0.030), Ferritin (*p* = 0.002) and LDH (*p* = 0.019) values were statistically significantly high in the non-survivor group. The mortality rate of the patients who had IMV was also statistically significantly high (*p* < 0.001) compared to patients who had HFNC and/or NIMV. Albumin (*p* = 0.032) value and the rate of treatment with HFNC and/or NIMV (*p* = 0.002) were statistically significantly low in non-survivor group compared to the survivor group. The cut-off value of the prognosis power of the inflammatory parameters was determined with ROC analysis (Fig. 1, Table 2). Cut-off values determining mortality, sensitivity, specificity, NPV, PPV, accuracy rates of inflammatory parameters (CRP, PCT, Ferritin, WBC) was presented at Table 3.

**Table 2:** Areas under the ROC curve (AUC) obtained for cut-off value analysis in detecting the mortality

Area Under the Curve			
Test Result Variable(s)	Area	Asymptotic 95% Confidence Interval	
CRP	0.640	0.529	0.750
PCT	0.621	0.510	0.731
Ferritin	0.675	0.580	0.771
WBC	0.563	0.462	0.663

**Table 3:** Inflammatory parameters. Cut-off values determining mortality, sensitivity, specificity, positive predictive value (PPV), negative predictive value (NPV), accuracy rates

Test Result Variable(s)	Cut-off Value	Sensitivity	Specificity	PPV	NPV	Accuracy Rates
**CRP**	**≥96.5**	72.1	58.8	87.8	33.9	69.5
**PCT**	**≥0.31**	72.9	52.9	86.4	32.1	70.0
**Ferritin**	**≥338**	64.5	60.6	87.3	29	63.4
**WBC**	**≥10,545**	54.0	50.0	81.5	21.0	53.2

The evaluation of the risk factors for mortality which was conducted with univariate logistic regression analysis in a model with variables *p* < 0,250 showed that age, CRP (≥96.5), PCT (≥0.31) Ferritin (≥338), IMV were the most significant prognostic parameters for mortality (*p* = 0.011 *p* = 0.001 *p* = 0.002 *p* = 0.004) (Table 4).

**Table 4:** Mortality risk factors - univariate Logistic Regression Analysis

		***P***	OR	%95 CI
**DEMOGRAPHY**	Age	***0.046***	1.096	1.001-1.198
	Age (Ref:80-90 age) > 90 age	0.355	1.824	0.511-6.510
	Sex (Ref:Male) Female	0.678	1.177	0.545-2.538
**COMORBIDITY**	DM	0.981	0.990	0.444-2.207
	HT	0.573	0.802	0.372-1.728
	CAD	0.946	1.029	0.452-2.339
	COPD	0.334	0.639	0.257-1.587
	Other	0.164	1.722	0.801-3.703
**LABORATORY VALUES**	CRP	***0.017***	1.006	1.001-1.011
	CRP (≥96.5)	***0.001***	3.700	1.702-8.043
	PCT	0.779	1.000	1.000-1.000
	PCT (≥0.31)	***0.005***	3.020	1.399-6.519
	Ferritin	***0.019***	1.001	1.000-1.002
	Ferritin (≥338)	***0.010***	2.794	1.280-6.099
	WBC	0.299	1.000	1.000
	WBC (≥10,545)	0.679	1.172	0.553-2.482
	D-dimer	0.834	1.000	1.000-1.000
	Troponin	0.351	1.000	1.000-1.001
	Lactate	0.119	1.236	0.947-1.613
	Albumin	0.096	0.963	0.922-1.007
	Urea	0.359	1.003	0.997-1.009
	Creatinine	0.570	1.095	0.801-1.497
	ALT	0.261	1.008	0.994-1.022
	AST	0.084	1.013	0.998-1.029
	PaO_2_	0.692	0.999	0.995-1.003
	PaCO_2_	0.836	1.002	0.979-1.026
	LDH	0.075	1.002	1.000-1.004
	Lymphocyte	0.770	1.000	1.000-1.000
**TREATMENT**	IMV	***< 0.001***	**197.2**	**49.88-779.58**
	HFNC and/or NIMV	***0.003***	0.296	0.131-0.666
	HDF	0.107	0.336	0.089-1.264
	Anti-cytokines	0.552	0.478	0.042-5.434
	Plasma	0.261	0.350	0.056-2.184
**COMPUTED TOMOGRAPHY**	No Infiltration	0.157		
	Unilateral Infiltration	0.437	1.636	0.473-5.661
	Bilateral Infiltration	0.056	2.392	0.978-5.849

Bold and italic values are statistically significant (*p* < 0.05)

## Discussion

The effects of clinical characteristics and the intensive care treatment methods on the mortality of Covid-19 patients aged 80 years and older were investigated in our study. During the Covid-19 pandemic elderly patients have higher rates of intensive care admission and mortality when compared with younger patients. The course of hospitalization, intensive care therapy and outcomes of the Covid-19 patients older than 65 years of age were investigated in previous studies [2–5]. However, only a few reports have analyzed the clinical course and characteristics of Covid-19 patients aged 80 years age and older [2, 3].

In our study, mortality rate was very high (%80.5) and mortality rates among admitted patients increased as the age increased (mean age of non-survivor group was 86 vs 83 in survivor group). High levels of CRP, PCT, ferritin and having IMV are detected as poor outcome markers.

In previous studies, higher mortality risks have been reported in men than in women. Since the density of immune-related genes and regulatory elements is higher in X-chromosome, sex may influence the infectious severity of SARS-CoV-2 [8]. However, in our study, despite higher number of male patients (*n* = 102), the mortality did not differ among genders. This can be explained by the fact that all patients were very elderly and frail.

In comparison with the recently published studies [3, 9], in our study, the duration of stay in ICU for both groups was short (mean 7 days). The reason for this comparatively short length of stay in ICU is that all patients with nasal cannula or reservoir mask were followed up in another unit in emergency department. The patients were admitted to ICU only when there was a need of intubation, HFNC and/or NIMV.

Laboratory tests were analyzed in many previous studies to determine the severity of the Covid-19 disease [3, 9]. Those studies have revealed that increased levels of CRP, PCT, D-dimer, troponin I, ferritin, ALT, AST, urea, creatinine, LDH occur in the severe manifestation of the Covid-19 disease. In our study similar to the previous studies, CRP, PCT, ferritin and LDH values were statistically high (*p* = 0,011 *p* = 0,030 *p* = 0,002 *p* = 0,019) in the non-survivor group. To determine the cut-off values of the inflammatory markers we used the ROC analysis and high levels of CRP, PCT and ferritin were found as indicators of poor outcome. Higher PCT implies a more severe condition of co-infection in Covid-19 patients.

In a previous study, lymphopenia (31.4%), increased D-dimer (38.1%), depressed albumin (36.2%), elevated LDH (41.0%), and a high level of CRP (79.0%) were common among elderly patients with Covid-19 [10]. In our study the albumin level of the non-survivor group was statistically significantly low (*p* = 0,032). D-dimer elevation is a considerable marker in worsening of the prognosis by coagulopathy and embolic incidents. In our study, D-dimer level of the non-survivor group was high even though it was not statistically significant. Li et al. [8] have shown that lymphopenia was a predictor of severe Covid-19. While the specific mechanism of lymphopenia has not been revealed, it is postulated that SARS-CoV particles targeted lymphocytes and destroyed its cytoplasmic components, thus causing a reduction of T cells. In our study, lymphocyte level of the non-survivor group was low compared to the survivor group although it was not statistically significant.

In the study published by Li et al., glucocorticoid, increased neutrophil and LDH were stated as predictive indicators for IMV [8]. Similar studies have suggested that increased level of LDH may be associated with the severity of Covid-19 disease. High LDH levels is considered in correlation with lung damage. In line with the previous studies, in our study in the non-survivor group the LDH levels were statistically significantly higher compared to survivor group (*p* = 0.019).

Pre-existing health conditions were determined as indicators of poor prognosis. According to the current information, the mortality rate was significantly higher among the elderly with HT, DM, CAD and malignancy [11, 12]. A critical course of the disease was observed more commonly in antihypertensive drug receivers. In recently published studies, it has been reported that the administration of angiotensin converting enzyme inhibitors and angiotensin II receptor blocker increased ACE2 receptor expression and the disease progressed severely [11, 13]. Similarly, in our study, the number of hypertensive patients was high (*n* = 100). Besides, we observed that the mortality rate was not statistically significantly high in patients with comorbities but the number of hypertensive patients was very high in the non-survivor group (*n* = 79).

Chest CT imaging has been widely used in the diagnosis of the Covid-19 pneumonia. On the CT, the diagnosis of Covid-19 pneumonia was generally made upon the appearance of ground-glass opacities [14]. In our study, the mortality of the patients who have bilaterally infiltrated pneumonia was high but CT imaging was not found as a statistically significant predictor for the mortality.

Studies which discuss mortality rates of Covid-19 patients aged 80 years and older are even more rare [15]. Based on current data, the mean for the case fatality rate for adults aged under 60 is estimated to be less than 0.2%, while for those aged over 80 this rate is 9.3% [11]. In a recent study, it was stated that the fatality rate for Covid-19 patients on ventilators aged 80 and older was 90% [4]. Similarly, during an influenza pandemic being 85 years and older was an exclusion criterion for ventilation [16]. Clarfield M et al. in their study concluded that for care of older persons during a pandemic, alternative treatment methods should be considered than mechanic ventilator therapy as this could not prevent mortality [5]. In our study, due to rapidly created new Covid-19 intensive care units and following ethical concerns no selection criteria (as old age or any comorbidity) was applied and all conventional treatments were applied to our patients. Consequently, a significant data about Covid-19 patients aged 80 years and older who received intensive care treatment was collected. Accordingly, in our study, ICU mortality rate was found 80.5% and only 5 patients who received IMV therapy could survive. In the review of Cavayas et al., where early experience with critically ill patients with Covid-19 in Montreal at *Sacre-Coeur de Montreal* Hospital was investigated: it was shown that mortality was 25, and 21% in the IMV patients. It was also stated that mortality rate was 82% in patients older than 80 years old [17]. The application of HFNC provides a better oxygenation by heating and moistening the oxygen with high flow. HFNC has lower transpulmonary pressures compared to IMV and causes less lung damage and it is a very important feature especially for elderly patients whose frailty is high [18]. In our study, it was found that the mortality rate was lower in patients who underwent HFNC and/or NIMV treatments compared to patients who had IMV.

In the study of Nabors C et al. patients 80 years or older requiring ICU care had a high mortality (57%) and mortality rate among patients who developed a need for RRT was %100.In this study it was also revealed that, 21% of the patients was received convalescent plasma and the mortality rate was found as 47% [3]. In our study, among the 10 patients who had HDF only 4 patients survived. Besides, among the 5 patients who received plasma therapy only 2 of them survived.

Clinical studies although limited in sample size and heterogeneity of the experimental design, show that the use of anti-IL-6 mAbs in Covid-19 patients has some positive effect on the improvement [19]. Those experimental therapies in elderly Covid-19 patients may ultimately worsen the outcomes [3]. We used anti-cytokine (tocilizumab) in the treatment of 3 patients and among them only 1 patient survived.

Our study has certain limitations. Firstly, our study is a retrospective study and it contains only the patients of a single hospital. Another limitation is that full medical history of the patients could not be exactly accessed due to the lack of communication caused by the pandemic conditions as well as the cognitive deficiency because of the old age of patients and their relatives. Finally, the number of patients received convalescent plasma and anti-cytokine treatments was very low.

## Conclusions

Our study showed that, ICU treatments may be beneficial for the Covid-19 patients aged 80 years and older. Increased old age, high levels of CRP, PCT, ferritin, and having IMV are detected as poor outcome markers. As a result, we emphasize that ICU treatments should also be considered as an option for the elderly Covid-19 patients.

## Data Availability

The datasets used and/or analysed during the current study available from the corresponding author on reasonable request.

## Abbreviations

ACE: Angiotensin-converting enzyme
ALT: Alanine aminotransferase
Anti-IL-6 mAb: Anti-interleukin monoclonal antibody
AST: Aspartate aminotransferase
CAD: Coronary artery disease
COPD: Chronic obstructive pulmonary disease
COVID-19: Coronavirus disease 2019
CRP: C-reactive protein
CT: Computed tomography
DM: Diabetes Mellitus
FiO_2_: Fraction of inspired oxygen
HDF: Hemodiafiltration
HFNC: Nasal high flow cannula
HT: Hypertension
ICU: Intensive care unit
IMV: Invasive mechanical ventilation
IQR: Interquartile range
LDH: Lactate dehydrogenase
NIMV: Non-invasive mechanical ventilation
PaO2: Partial pressure of oxygen
PaCO_2_: Partial pressure of carbon dioxide
PCT: Procalcitonin
ROC: Receiver Operating Characteristic
SARS-CoV2: Severe acute respiratory syndrome coronavirus 2
WBC: White blood cell
